# Effects of Low and High Doses of Aspirin on Inflammatory Markers in Diabesity Patients: A Quasi-Experimental Study

**DOI:** 10.7759/cureus.60659

**Published:** 2024-05-20

**Authors:** Mridu Singh, Rupita Kulshrestha, Vikram Singh, Anumesh K Pathak, Abhisek Kumar, Shivani Singh, Gopal K Bohra

**Affiliations:** 1 Medicine, Dr. Ram Manohar Lohia Institute of Medical Sciences, Lucknow, IND; 2 Obstetrics and Gynaecology, Dr. Ram Manohar Lohia Institute of Medical Sciences, Lucknow, IND; 3 Biochemistry, Dr. Ram Manohar Lohia Institute of Medical Sciences, Lucknow, IND; 4 Medicine, All India Institute of Medical Sciences, Jodhpur, IND

**Keywords:** type 2 diabetes mellitus, obese, interleukin -6, hs-crp, diabesity, aspirin

## Abstract

Introduction

The intertwined nature of obesity and diabetes, termed diabesity, is a significant health concern. Aspirin has been recognized for its potential in mitigating inflammation-related health issues, a key concern in managing diabesity. However, the optimal aspirin dosage and its impact on specific inflammatory markers, viz. high-sensitivity C-reactive protein (hs-CRP) and interleukin (IL)-6, over time remain a subject of ongoing research.

Objective

This study investigated the effects of different doses of aspirin (150mg and 300mg) on the levels of hs-CRP and IL-6 over a period of 6 months.

Methods

This cross-sectional observational quasi-experiment study involved 125 confirmed type-2 diabetes mellitus (T2DM) patients with obesity aged ≥40 years. Blood samples were collected for analyzing hs-CRP and IL-6 levels. Demographics and clinical characteristics, such as BMI, waist-hip ratio, blood parameters, fasting blood sugar (FBS), and hs-CRP, were analyzed.

Results

At baseline, both the 150 mg and 300 mg aspirin dose groups had similar median levels of hs-CRP. After two months, there was no significant difference (p=0.150). However, by six months, the 150mg dose group had a significantly higher median hs-CRP than the 300 mg dose group (p=0.003). The 150 mg dose group had a significantly higher median level of IL-6 levels at baseline (median; 40.0) compared to the 300 mg dose group (median; 2.27, p<0.0001). After two months, the levels of IL-6 in both groups were similar (median; 2.27 and 2.23 respectively, p<0.0001). By the end of six months, the groups had no significant difference (median; 0.53 and 2.22 respectively, p=0.128).

Conclusion

The dose of aspirin may significantly impact the levels of hs-CRP and IL-6 over time, with the effects being more pronounced after six months of treatment. These findings suggest that aspirin, a commonly used and cost-effective medication, could potentially be leveraged in a more targeted manner to manage inflammation (CRP and IL-6 levels) in individuals with diabesity.

## Introduction

The field of medicine has long recognized the potential of aspirin in mitigating inflammation-related health issues, a key concern in managing diabesity [1]. Diabesity, a complex condition characterized by the coexistence of obesity and type 2 diabetes mellitus (T2DM), is a growing global health concern. Diabesity is linked to chronic low-grade inflammation and activation of immune systems that contribute to the development of obesity-related insulin resistance (IR) and T2DM [2]. Various experimental and clinical studies have unequivocally demonstrated that adipose tissue, liver, muscle, and pancreas exhibit inflammation in individuals with obesity and T2DM. Macrophages infiltrate these tissues in animal models of obesity and diabetes and with metabolic syndrome or T2DM [3,4].

Serum inflammatory markers, including interleukin-6 (IL-6) and high-sensitivity C-reactive protein (hs-CRP), are increased in obese individuals, suggesting that diabesity can be characterized as an inflammatory condition [5,6]. IL-6 and hs-CRP are physiological markers of subclinical systemic inflammation that have been linked to hyperglycemia, insulin resistance, and T2DM [7,8].

Aspirin, a non-steroidal anti-inflammatory drug, has been suggested as a potential therapeutic agent for diabesity due to its anti-inflammatory properties [9]. Aspirin inhibits cyclooxygenase (COX), an enzyme that produces prostaglandins, which contributes to inflammation. By suppressing the production of prostaglandins, aspirin results in the inactivation of COX, and consequently, this prevents inflammation. However, the optimal dosage and duration of aspirin administration for maximum anti-inflammatory effect in diabesity patients remain unclear.

The available literature provides limited insights into the effects of different doses of aspirin with time intervals on inflammatory markers in diabesity patients. Most existing studies have focused on the effects of aspirin in either diabetes or obesity, but not the combined condition of diabesity [10,11]. Furthermore, studies have not adequately explored the impact of different doses of aspirin over varying durations.

Studies have shown that aspirin inhibits cyclooxygenase (COX), an enzyme responsible for the production of prostaglandins that contribute to inflammation. By suppressing prostaglandin production, aspirin inactivates COX, thereby preventing inflammation. Low-dose aspirin may be effective in reducing obesity-induced inflammation. However, the optimal dosage of aspirin for treating inflammation caused by obesity is not yet well established. A study conducted on mice demonstrated that a low dose of aspirin (100 mg/day for 11 weeks) inhibited the expression of IL-6 and TNFα in adipocytes, reduced adipogenesis, and decreased adipocyte inflammation [12]. Conversely, a higher dose of aspirin can reduce inflammation by further inhibiting COX [13]. We hypothesized that low and high doses of aspirin will significantly reduce hs-CRP and IL-6 levels in diabesity patients and that these reductions will be more significant over a longer duration of administration. This study aimed to fill this research gap by observing the changes in two inflammatory markers, hs-CRP and IL-6, in diabesity patients following the administration of low and high doses of aspirin over short and long durations.

## Materials and methods

Study design and settings

This study was a cross-sectional observational quasi-experiment study. The study population consisted of 125 confirmed T2DM patients with obesity aged ≥40 years. The initial phase of this study involved a comprehensive examination of a patient cohort of 623 individuals. The selection criteria for this group were stringent, focusing on patients whose HbA1C levels did not decrease by more than 1%, and whose BMI status remained unchanged, specifically those who did not transition from an obese to an overweight classification. Following this initial assessment, a subset of 125 cases was meticulously chosen for a more detailed evaluation. Further, the study subjects were divided into three groups based on their BMI and T2DM status: normal weight (n=31), overweight (n=48), and obese (n=46).

This selection was driven by the objective of investigating the inflammatory response in these patients. The chosen cases presented a unique opportunity to delve deeper into the complexities of the inflammatory response within the context of stable HbA1C and BMI conditions. Patients who used any antioxidant substance, insulin, anticoagulants, steroids, or non-acetylsalicylic acid anti-inflammatory drugs for treatment and had severe renal and hepatic failure, malignant tumors, and diabetes with acute ketoacidosis and hyperosmolar coma were excluded from the study.

The present study protocol was approved by the ethics committee of All India Institute of Medical Sciences, Jodhpur, India (Ref No. AIIMS/RES/(02)2016/338). Also, all the study participants were enrolled after getting the written informed consent.

Measurements and treatment protocol

Body weight, height, and waist and hip circumferences of the patients were measured. Obesity was defined as a BMI ≥ 30 kg/m^2^, as per the ADA criteria for the Asian population [14]. According to WHO criteria, the waist-hip ratio (WHR) was calculated by dividing waist circumference (cm) by hip circumference (cm). Patients were explored with diet, exercise, oral medication, and either 150mg or 300mg of aspirin for 2 and 6 months, respectively.

Blood sample collection and analysis

Blood samples from all the subjects were collected in a plain and ethylenediaminetetraacetic acid (EDTA) vial. Serum was obtained after centrifugation of a plain vial at 3500 rpm for 15 minutes. The serum was used to analyze the level of hs-CRP and IL-6.

Biochemical analysis

Fasting blood sugar (FBS) and hs-CRP were analyzed using commercially available reagents on a fully automated analyzer (Beckman Coulter, AU480), and HbA1C was analyzed from an EDTA sample on Variant Turbo (Bio-Rad). IL-6 was measured using chemiluminescence in serum samples on a fully automated analyzer (Beckman Coulter, Access 2).

Statistical analysis

The normality of the data was assessed using the Kolmogorov-Smirnov test. Data were presented as median and quartile ranges (25th and 75th) and analyzed using SPSS 21 IBM software (IBM Corp. Released 2012. IBM SPSS Statistics for Windows, Version 21.0. Armonk, NY: IBM Corp.). Group comparisons were performed using the Chi-square test and Mann-Whitney test. The Wilcoxon paired t-test was used to compare follow-up data for hs-CRP and IL-6. The Pearson correlation coefficient was employed to analyze the relationship between variables. A p-value of less than 0.05 was considered statistically significant.

## Results

Diabetic markers and inflammatory markers in diabesity patients

Table 1 compares baseline characteristics and various diabetic metrics across three different BMI groups: normal (n=31), overweight (n=48), and obese (n=46). In terms of gender distribution, the mean age in the obese group was 40.97±8.20 years, 38.60±7.80 years in the overweight, and 37.78±9.34 years in the normal group; there were no statistically significant differences (p>0.05) between these groups. The median BMI values were significantly different across the groups, with the obese group having the highest median BMI (33.96 kg/m2), followed by the overweight (27.12 kg/m2) and normal (22.02 kg/m2) groups (p<0.0001).

**Table 1:** Baseline characteristics in diabesity patients. BMI: Body mass index, hs-CRP: high sensitivity- C-reactive protein, IL-6: Interleukin-6, N vs. O: Normal versus Overweight, N vs. Ob: Normal versus Obese, O vs. Ob: Overweight versus Obese. Mann-Whitney test was used to calculate the p-value. p-value <0.05 was considered as statistically significant.

Variables		Normal (n=31)	Overweight (n=48)	Obese (n=46)	p-value (N vs. O)	p-value (N vs. Ob.)	p-value (O vs. Ob.)
Gender, N (%)	Male	19 (61.29)	31 (64.58)	27 (58.70)	0.766	0.819	0.557
	Female	12 (38.71)	17 (35.42)	19 (41.30)			
Age (Years) Mean±SD		37.78±9.34	38.60±7.80	40.97±8.20	0.674	0.117	0.154
BMI (kg/m^2^) Median (Q1-Q3)		22.02 (22.05-24.16)	27.12 (26.08-28.48)	33.96 (31.53-37.42)	<0.0001	<0.0001	<0.0001
FBS (mg/dL) Median (Q1-Q3)		134.00 (127.0-146.0)	151.00 (146.0-181.5)	183.40 (150.65-284.40)	0.027	0.001	0.024
HbA1c (%) Median (Q1-Q3)		6.74 (6.55-8.35)	7.00 (6.0-8.35)	9.00 (7.60-12.00)	0.557	0.001	0.001
hs-CRP (mg/L) Median (Q1-Q3)		3.00 (1.10-7.30)	5.20 (2.30-13.70)	12.20 (5.90-23.00)	0.020	<0.0001	0.001
IL-6 (pg/mL) Median (Q1-Q3)		9.70 (2.30-40.0)	9.70 (2.30-48.00)	12.10 (2.30-70.00)	0.202	<0.0001	<0.0001

The median values of FBS levels in the normal, overweight, and obese groups were 134.00 mg/dL (p=0.024), 151.00 mg/dL (p=0.001), and 183.40 mg/dL (p=0.027), respectively. The normal weight group had a median HbA1c level of 6.74%, the overweight group had a median HbA1C level of 7.00%, and the obese group had the highest median HbA1c level of 9.00% (p=0.001). The hs-CRP levels were also different among the groups. The normal weight group had a median level of 3.00 mg/L(p=0.020), the overweight group had a median level of 5.20 mg/L (p<0.0001), and the obese group had the highest median level of 12.20 mg/L (p=0.001), respectively. Similarly, IL-6 levels in the normal weight group were at a median level of 9.70 pg/mL, the overweight group had a similar median level of 9.70 pg/mL, and the obese group had a slightly higher median level of 12.10 pg/mL. The differences in IL-6 levels between the groups were statistically significant (p<0.0001).

Aspirin-induced dynamic changes in hs-CRP levels in diabesity patients

For the normal group receiving 150 mg, the baseline median of hs-CRP levels was 3.00 mg/L, which decreased to 1.10 mg/L at 2 months (p= 0.001) and further decreased to 0.20 mg/L at 6 months (p=0.001) (Table 2). The overweight group receiving 150 mg had a baseline median of 5.20 mg/L, which decreased to 1.90 mg/L at 2 months, and further reduced to 0.6 mg/L at 6 months (p<0.0001).

**Table 2:** The hs-CRP levels for 150 mg and 300 mg doses of aspirin The values are represented as median (Q1-Q3). The tables show the levels of hs-CRP (mg/L) at baseline, 2 months, and 6 months for normal, overweight, and obese groups. P values indicate the significance of the difference between the time points.

BMI groups	Dose (mg)	Baseline	2 months	6 months	p-value (Baseline vs. 2 months)	p-value (Baseline vs. 6 months)	p-value (2 months vs. 6 months)
Normal (n=31)	150	3.0 (1.10-7.30)	1.10 (0.30-3.40)	0.2 (0.00-1.10)	0.001	<0.0001	<0.0001
Overweight (n=48)	150	5.20 (2.30-13.70)	1.90 (1.10-7.20)	0.6 (0.10-1.40)	<0.0001	<0.0001	<0.0001
Obese (46)	150	12.20 (5.90-23.00)	4.10 (1.20-6.80)	2.1 (0.20-3.00)	<0.0001	<0.0001	<0.0001
Normal (n=31)	300	2.37 (1.28-5.40)	1.20 (0.23-2.25)	0.3 (0.11-1.15)	<0.0001	<0.0001	<0.0001
Overweight (n=48)	300	6.96 (0.34-1.30)	2.60 (1.10-4.56)	0.28 (0.12-0.98)	0.001	<0.0001	<0.0001
Obese (46)	300	14.12 (0.85-22.08)	3.10 (1.15-5.99)	0.22 (0.12-2.15)	<0.0001	<0.0001	<0.0001

Similarly, the obese group receiving 150 mg had a baseline median of 12.20 mg/dL, which decreased to 4.10 mg/dL at 2 months and further decreased to 2.10 mg/dL at 6 months (p=0.0001) for all comparisons. For the normal group receiving 300 mg, the baseline median was 2.37 mg/dL, which decreased to 1.20 mg/dL at 2 months and further decreased to 0.30 mg/dL at 6 months (p<0.0001 for all comparisons). The overweight group receiving 300 mg had a baseline median of 6.96 mg/dL, which decreased to 2.60 mg/dL at 2 months and further reduced to 0.28 mg/dL at 6 months. The p-value was 0.001 for baseline vs. 2 months, p=0.0001 for baseline vs. 6 months, and p=0.0001 for 2 months vs. 6 months. The obese group receiving 300 mg had a baseline median of 14.12 mg/dL, which decreased to 3.10 mg/dL at 2 months and further reduced to 0.22 mg/dL at 6 months (p= 0.0001).

Aspirin-induced dynamic changes in IL-6 levels in diabesity patients

For the 150 mg dose, all BMI groups demonstrated a significant decrease in IL-6 from the baseline to the 2-month mark and then to the 6-month mark (with all p<0.0001) (Table 3). Specifically, the normal group’s values decreased from a baseline of 9.70 pg/mL to 2.20 pg/mL at 2 months (p-value <0.0001 compared to baseline) and further to 2.10 pg/mL at 6 months (p<0.0001 compared to baseline and p-value <0.0001 compared to 2 months). The overweight group showed a decrease from a baseline of 9.70 pg/mL to 2.30 pg/mL at 2 months (p <0.0001 compared to baseline) and further to 0.50 pg/mL at 6 months (p<0.0001 compared to baseline and p<0.0001 compared to 2 months). The obese group’s values decreased from a baseline of 12.10 pg/mL to 2.30 pg/mL at 2 months (p<0.0001 compared to baseline) and slightly to 2.20 pg/mL at 6 months (p<0.0001 compared to baseline and p<0.0001 compared to 2 months).

**Table 3:** IL-6 levels for 150 mg and 300 mg doses of aspirin. The values are represented as median (Q1-Q3). The table show the levels of IL-6 (pg/mL) at baseline, 2 months, and 6 months for normal, overweight, and obese groups. P values indicate the significance of the difference between the time points. The dose column suggests the dose of aspirin used in the study.

BMI groups	Dose (mg)	Baseline	2 months	6 months	p-value (Baseline vs. 2 months)	p-value (Baseline vs. 6 months)	p-value (2 months vs. 6 moths)
Normal (n=31)	150	9.70 (2.30-40.0)	2.20 (2.20-2.30)	2.10 (0.55-2.30)	<0.0001	<0.0001	<0.0001
Overweight (n=48)	150	9.70 (2.30-48.00)	2.30 (2.20-20.0)	0.50 (0.50-2.30)	<0.0001	<0.0001	<0.0001
Obese (n=46)	150	12.10 (2.30-70.00)	2.30 (2.20-20.0)	2.20 (0.50-2.30)	<0.0001	<0.0001	<0.0001
Normal (n=31)	300	2.27 (2.25-9.70)	2.30 (2.20-2.27)	2.20 (0.50-2.27)	0.001	<0.0001	0.012
Overweight (n=48)	300	2.28 (2.23-21.70)	2.30 (0.17-2.30)	1.37 (0.50-2.31)	<0.0001	0.001	0.043
Obese (n=46)	300	7.10 (2.27-40.00)	2.23 (2.23-2.27)	2.23 (0.50-2.27)	<0.0001	<0.0001	<0.0001

When the dose was increased to 300 mg, all BMI groups also showed a significant decrease in their values from the baseline to the 6-month. The normal group’s values slightly increased from a baseline of 2.27 pg/mL to 2.3 pg/mL at 2 months (p=0.001 compared to baseline) and then decreased to 2.20 pg/mL at 6 months (p<0.0001 compared to baseline and p=0.012 compared to 2 months). The overweight group’s values slightly increased from a baseline of 2.28 pg/mL to 2.30 pg/mL at 2 months (p<0.0001 compared to baseline) and then decreased to 1.37 pg/mL at 6 months (p=0.001 compared to baseline and p-value 0.043 compared to 2 months). The obese group’s values decreased from a baseline of 7.10 pg/mL to 2.23 pg/mL at 2 months (p<0.0001 compared to baseline) and remained the same at 6 months (p<0.0001 compared to baseline and p<0.0001 compared to 2 months). These results suggest that both doses significantly affected the values across all BMI groups over the 6 months.

Comparison between dose-dependent and time-dependent effect of aspirin on IL-6 and hsCRP levels

**Figure 1:**
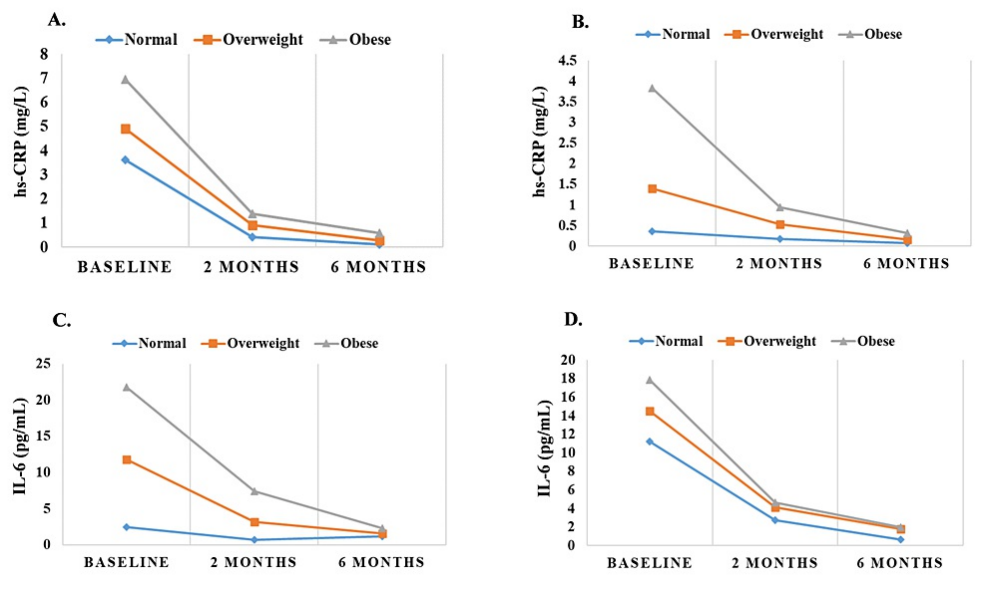
Status of hs-CRP and IL-6 levels on the basis of obesity at different time intervals and different doses of aspirin. A) after the onset of 150mg hs-CRP levels, B) after onset of 300mg hs-CRP levels, C) after onset of 150mg IL-6 levels, D) after onset of 300mg IL-6 levels.

Comparison between the dose-dependent and time-dependent effect of aspirin on IL-6 and hsCRP levels were depicted in Table 4 and Figure 1. The study examined the effects of two different doses of aspirin, 150 mg and 300 mg, on the levels of hs-CRP (mg/L) and IL-6 (pg/mL) over a period of 6 months. At the start of the study, both groups had similar median levels of hs-CRP (median levels at 7.31 and 7.30, respectively, p=0.875). After 2 months, there was no significant difference (median levels at 2.40 and 2.20 respectively, p=0.150). However, by the 6-month mark, the 150mg dose group had a significantly higher median level of hs-CRP (median: 1.10) than the 300mg dose group (median: 0.23, p=0.003).

**Table 4:** Comparison between the dose-dependent and time-dependent effect of aspirin on IL-6 and hsCRP levels. BMI: Body mass index, WHR: Waist hip ratio, hs-CRP: high sensitivity- C-reactive protein, IL-6: Interleukin-6.

Variables	150 mg dose of aspirin (n=125) Median (Q1-Q3)	300 mg dose of aspirin (n=89) Median (Q1-Q3)	p-value
hs-CRP (mg/L) (Baseline)	7.31 (1.92-16.95)	7.30 (3.04-14.08)	0.875
hs-CRP (mg/L) (2 months)	2.40 (1.00-7.60)	2.20 (1.10-4.35)	0.150
hs-CRP (mg/L) (6 months)	1.10 (0.20-2.70)	0.23 (0.12-1.16)	0.003
IL-6 (pg/mL) (Baseline)	40.00 (2.27-70.00)	2.27 (2.27-40.00)	<0.0001
IL-6 (pg/mL) (2 months)	2.27 (2.23-20.00)	2.23 (2.22-2.27)	<0.0001
IL-6 (pg/mL) (6 months)	0.53 (0.50-2.27)	2.22 (0.50-2.25)	0.128

In terms of IL-6 levels (pg/mL), the 150mg dose group had a significantly higher median level at baseline (median: 40.00) compared to the 300mg dose group (median: 2.27, p<0.0001). After 2 months, the levels in both groups were similar (median; 2.27 and 2.23 respectively, p<0.0001). By the end of 6 months, the groups had no significant difference (median; 0.53 and 2.22, respectively, p=0.128).

Correlation between baseline hs-CRP, IL-6, and BMI in diabetic patients

**Figure 2:**
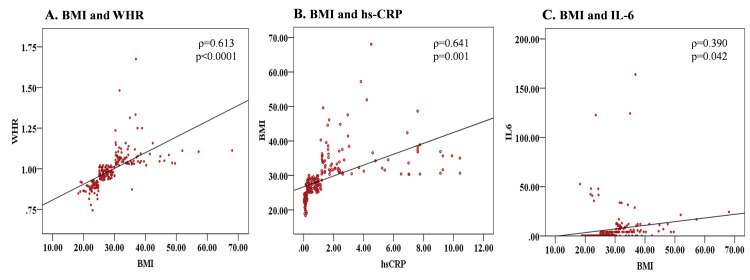
Correlation of BMI with WHR, hs-CRP, and IL-6 levels. ρ= Spearman correlation coefficient, p= p-value

BMI is found to have a strong positive correlation with waist-hip ratio (WHR) (ρ=0.613, p<0.0001), hs-CRP (ρ=0.641, p=0.001), and baseline IL-6 (ρ=0.390, p=0.042) (Figure 2). WHR also exhibits a strong positive correlation with hs-CRP (ρ=0.648, p<0.0001), but interestingly, it shows a negative correlation with baseline IL-6 (ρ=-0.180, p=0.008). The hs-CRP and baseline IL-6 also correlate positively (ρ=0.273, p<0.0001).

## Discussion

The results of this study provide valuable insights into the impact of aspirin dosage on inflammatory markers in patients with diabesity, a condition characterized by the co-existence of obesity and diabetes. Our study found significant changes in the levels of hs-CRP and IL-6 over a period of 6 months in response to two different doses of aspirin, 150mg and 300mg.

Our study tested the hypothesis that aspirin (acetylsalicylic acid) reduces low-grade inflammation in patients with diabesity. The results supported this hypothesis, showing that aspirin significantly decreased the levels of hs-CRP and IL-6, markers of inflammation, in diabesity patients. To our knowledge, this is the first study in North India to investigate the impact of aspirin on inflammation in diabesity patients.

Aspirin, one of the most widely used medications for its anti-platelet action, is believed to work by blocking the formation of arachidonic acid-derived inflammatory mediators [15]. This concept aligns with data from genetic models suggesting that salicylates can treat diabetes by reversing chronic inflammation [16].

The study found no significant difference in the mean age across the BMI groups. However, there were significant differences in the median BMI values, with the obese group having the highest median BMI, followed by the overweight and normal groups. This is consistent with the definitions of these groups.

In terms of diabetic metrics, the study found that FBS levels, HbA1c levels, and hs-CRP levels were highest in the obese group, followed by the overweight and normal groups. Interestingly, the IL-6 levels were similar for the normal and overweight groups but slightly higher for the obese groups. These findings suggest that obesity is associated with higher levels of these diabetic metrics, indicating a higher risk of diabetes and inflammation in obese individuals.

The present study also investigated the impact of aspirin dosage on hs-CRP levels in these individuals. It was found that both the 150 mg and 300 mg doses of aspirin led to a significant decrease in hs-CRP levels from baseline to 2 and further to 6 months in all BMI groups. This suggests that aspirin positively reduces inflammation, as indicated by the decrease in hs-CRP levels, in individuals with diabesity, regardless of their BMI. Our findings align with a previous study that examined the effects of a daily 300mg aspirin dosage on hs-CRP and IL-6 levels in 40 men with coronary artery disease. The results of this study provide significant evidence supporting the anti-inflammatory effects of aspirin treatment in patients. The observed decrease in CRP and IL-6 levels in the aspirin-treated group compared to the placebo group is noteworthy in our study. Specifically, CRP levels were significantly lower in the aspirin-treated group, with median levels of 1.0 (0.5-3.1) mg/L compared to 1.4 (0.5-4.1) mg/L in the placebo group (p< 0.05). Similarly, IL-6 levels were also significantly reduced in the aspirin-treated group, with median levels of 2.9 (2.5-3.4) pg/mL compared to 3.5 (3.2-4.6) pg/mL in the placebo group (p< 0.05). These findings suggest that aspirin treatment may effectively reduce inflammation in patients, as indicated by the lower levels of these inflammatory markers [17]. Moreover, low-dose aspirin may be effective in reducing obesity and inflammation induced by obesity. However, the ideal dosage of aspirin for treating inflammation caused by obesity is not yet well established. A study conducted on mice showed that a low dose of aspirin (100 mg/day for 11 weeks) led to inhibiting the expression of IL-6, and TNFα in adipocytes. Furthermore, aspirin reduces adipogenesis and adipocyte inflammation [12]. On the other hand, a higher dose of aspirin can reduce inflammation by inhibiting COX. Therefore, a higher dose of aspirin, such as 300 mg, may be effective in reducing inflammation and improving insulin sensitivity in individuals with diabesity [13,18].

In contrast, two earlier studies evaluated the effects of aspirin on CRP in healthy volunteers, in which no effects were found [19,20]. The adjusted for baseline hs-CRP and IL-6 levels showed high intra-individual variation, and were unable to assess the effect of aspirin on these inflammatory markers in diabesity patients. Our data suggest that aspirin has a more profound effect on reducing hs-CRP and IL-6; this aligns with larger prospective studies on primary prevention of cardiovascular events in diabetic patients, which have shown more beneficial outcomes with daily aspirin dosages higher than 100 mg, compared to studies using 100 mg or less of aspirin daily [21-24]. However, a direct comparative study on diabesity has yet to be conducted.

A significant correlation was observed between hs-CRP and IL-6 levels both before and after the completion of aspirin therapy. This correlation is likely attributable to the coexistence of acute and chronic inflammation. Acute inflammation is a temporary protective response, while chronic inflammation is a persistent condition triggered by various factors [25]. In overweight or obese individuals, infiltrating immune cells into adipose tissue can lead to chronic inflammation [26]. The relationship between obesity and inflammation is cyclical, with weight gain triggering inflammation and inflammation promoting further weight gain [27]. It is suggested that managing chronic, low-grade inflammation could be as vital as diet and exercise in addressing obesity [28,29]. Weight loss in overweight or obese individuals is a key factor in reducing pro-inflammatory markers [30].

The study's limitations include the possibility that the results may not accurately reflect the prevalence of diabetes in bigger populations or in other places due to its limited sample size and geographical location. The study cohort was selected using strict criteria, which may have limited its applicability to diabesity individuals with diverse characteristics. The cross-sectional approach limits causal linkages between aspirin dosage and inflammatory marker changes; longitudinal or randomized controlled trials might give better evidence. Only two aspirin dosage regimens were studied between 2 and 6 months, limiting dose-response relationships and optimal treatment duration. The study did not examine aspirin adherence and long-term effects beyond 6 months, potentially altering treatment outcomes. The study did not take into consideration potential confounding factors such as food, physical activity, or concurrent medications, all of which could have an impact on inflammation markers and treatment effects.

## Conclusions

Individuals with diabesity exhibit elevated levels of inflammatory markers, such as hs-CRP and IL-6, which are proportional to their BMI. Our study demonstrates that aspirin, a known anti-inflammatory drug, can effectively reduce these levels in obese individuals with T2DM. This finding is significant as it suggests a potential strategy for mitigating the complications associated with obesity and insulin resistance. The findings suggest that aspirin, a commonly used and cost-effective medication, could potentially be leveraged in a more targeted manner to manage inflammation in individuals with diabesity. The aspirin dose was found to significantly impact the levels of these markers, with the effects becoming more pronounced after 6 months of treatment. This suggests that long-term use of aspirin could have beneficial effects in managing inflammation in diabesity. However, much remains to be learned about the optimal use of aspirin in this context. Future research should aim to confirm these findings and explore their clinical implications, aiming to improve treatment strategies for individuals with diabesity.

## References

[1] Chen L, Magliano DJ, Zimmet PZ (2011). The worldwide epidemiology of type 2 diabetes mellitus--present and future perspectives. Nat Rev Endocrinol.

[2] Jehan S, Myers AK, Zizi F, Pandi-Perumal SR, Jean-Louis G, McFarlane SI (2018). Obesity, obstructive sleep apnea and type 2 diabetes mellitus: Epidemiology and pathophysiologic insights. Sleep Med Disord.

[3] Ni D, Smyth HE, Cozzolino D, Gidley MJ (2022). Integrating effects of human physiology, psychology, and individual variations on satiety-an exploratory study. Front Nutr.

[4] Hu FB, Li TY, Colditz GA, Willett WC, Manson JE (2003). Television watching and other sedentary behaviors in relation to risk of obesity and type 2 diabetes mellitus in women. JAMA.

[5] Wharton S, Sharma AM, Lau DC (2013). Weight management in diabetes. Can J Diabetes.

[6] Asghar A, Sheikh N (2017). Role of immune cells in obesity induced low grade inflammation and insulin resistance. Cell Immunol.

[7] Ni Y, Ni L, Zhuge F, Xu L, Fu Z, Ota T (2020). Adipose tissue macrophage phenotypes and characteristics: the key to insulin resistance in obesity and metabolic disorders. Obesity (Silver Spring).

[8] Tangvarasittichai S, Pongthaisong S, Tangvarasittichai O (2016). Tumor necrosis factor-α, interleukin-6, C-reactive protein levels and insulin resistance associated with type 2 diabetes in abdominal obesity women. Indian J Clin Biochem.

[9] Illán-Gómez F, Gonzálvez-Ortega M, Orea-Soler I (2012). Obesity and inflammation: change in adiponectin, C-reactive protein, tumour necrosis factor-alpha and interleukin-6 after bariatric surgery. Obes Surg.

[10] Kim KE, Heo JS, Han S (2018). Blood concentrations of lipopolysaccharide-binding protein, high-sensitivity C-reactive protein, tumor necrosis factor-α, and Interleukin-6 in relation to insulin resistance in young adolescents. Clin Chim Acta.

[11] Uemura H, Katsuura-Kamano S, Yamaguchi M, Bahari T, Ishizu M, Fujioka M, Arisawa K (2017). Relationships of serum high-sensitivity C-reactive protein and body size with insulin resistance in a Japanese cohort. PLoS One.

[12] Roy S, Bhowmik DR, Begum R, Amin MT, Islam MA, Ahmed F, Hossain MS (2022). Aspirin attenuates the expression of adhesion molecules, risk of obesity, and adipose tissue inflammation in high-fat diet-induced obese mice. Prostaglandins Other Lipid Mediat.

[13] Vane JR, Botting RM (2003). The mechanism of action of aspirin. Thromb Res.

[14] Alberti KG, Zimmet PZ (1998). Definition, diagnosis and classification of diabetes mellitus and its complications. Part 1: diagnosis and classification of diabetes mellitus provisional report of a WHO consultation. Diabet Med.

[15] Vane JR, Botting RM (1998). Anti-inflammatory drugs and their mechanism of action. Inflamm Res.

[16] Yuan M, Konstantopoulos N, Lee J, Hansen L, Li ZW, Karin M, Shoelson SE (2001). Reversal of obesity- and diet-induced insulin resistance with salicylates or targeted disruption of Ikkbeta. Science.

[17] Hovens MM, Snoep JD, Groeneveld Y, Frölich M, Tamsma JT, Huisman MV (2008). Effects of aspirin on serum C-reactive protein and interleukin-6 levels in patients with type 2 diabetes without cardiovascular disease: a randomized placebo-controlled crossover trial. Diabetes Obes Metab.

[18] Grosser T, Smyth E, FitzGerald G (2023). Pharmacotherapy of inflammation, fever, pain, and gout. Goodman & Gilman’s: The Pharmacological Basis of Therapeutics.

[19] Feng D, Tracy RP, Lipinska I, Murillo J, McKenna C, Tofler GH (2000). Effect of short-term aspirin use on C-reactive protein. J Thromb Thrombolysis.

[20] Feldman M, Jialal I, Devaraj S, Cryer B (2001). Effects of low-dose aspirin on serum C-reactive protein and thromboxane B2 concentrations: a placebo-controlled study using a highly sensitive C-reactive protein assay. J Am Coll Cardiol.

[21] Pradhan AD, Manson JE, Rifai N, Buring JE, Ridker PM (2001). C-reactive protein, interleukin 6, and risk of developing type 2 diabetes mellitus. JAMA.

[22] Steering Committee of the Physicians' Health Study Research Group (1989). Final report on the aspirin component of the ongoing Physicians' Health Study. N Engl J Med.

[23] Kassoff A, Buzney SM, McMeel JW (1992). Aspirin effects on mortality and morbidity in patients with diabetes mellitus. Early Treatment Diabetic Retinopathy Study report 14. ETDRS Investigators. JAMA.

[24] Sacco M, Pellegrini F, Roncaglioni MC, Avanzini F, Tognoni G, Nicolucci A (2003). Primary prevention of cardiovascular events with low-dose aspirin and vitamin E in type 2 diabetic patients: results of the Primary Prevention Project (PPP) trial. Diabetes Care.

[25] Ridker PM, Cook NR, Lee IM (2005). A randomized trial of low-dose aspirin in the primary prevention of cardiovascular disease in women. N Engl J Med.

[26] Oh DY, Walenta E, Akiyama TE (2014). A Gpr120-selective agonist improves insulin resistance and chronic inflammation in obese mice. Nat Med.

[27] Monteiro R, Azevedo I (2010). Chronic inflammation in obesity and the metabolic syndrome. Mediators Inflamm.

[28] Petelin A, Bizjak M, Černelič-Bizjak M, Jurdana M, Jakus T, Jenko-Pražnikar Z (2014). Low-grade inflammation in overweight and obese adults is affected by weight loss program. J Endocrinol Invest.

[29] Khanna D, Khanna S, Khanna P, Kahar P, Patel BM (2022). Obesity: a chronic low-grade inflammation and its markers. 2022.

[30] You T, Arsenis NC, Disanzo BL, Lamonte MJ (2013). Effects of exercise training on chronic inflammation in obesity : current evidence and potential mechanisms. Sports Med.

